# Modulatory Effect of Vitamin C on Hypoxia Induced Breast Cancer Stem Cells

**DOI:** 10.34172/apb.2023.073

**Published:** 2023-02-21

**Authors:** Masoumeh Kazemi, Soheila Montazersaheb, Mina Noroozpour, Safar Farajnia, Hojjatollah Nozad Charoudeh

**Affiliations:** ^1^Drug Applied Research Center, Tabriz University of Medical Sciences, Tabriz, Iran.; ^2^Department of Medical Genetics, Shahid Beheshti University of Medical Sciences, Tehran, Iran.; ^3^Molecular Medicine Research Center, Tabriz University of Medical Sciences, Tabriz, Iran.; ^4^Faculty of Materials Science and Engineering, Sahand University of Technology, Tabriz, Iran.

**Keywords:** Vitamin C, Hypoxia, MCF7, Cancer Stem Cell

## Abstract

**Purpose:**

Eliminating cancer stem cells (CSCs) is a challenge because of their enhanced resistance to anti-cancer drugs. Vitamin C, which is insufficient in patients with higher stages of cancer, has been gaining attention as a potential treatment for human malignancies. Hence this study aimed to analyze the effect of high-dose vitamin C treatment on the gene expression level of *HIF-1α*, *NF-κB1*, *BAX*, and *DNMT1* in the MCF7 cells undergoing hypoxia, as an inducer of CSCs characteristics. As a result, vitamin C could be possibly used as a promising therapeutic adjuvant.

**Methods:**

Here we first analyzed the breast CSC population alteration in MCF7 cells following hypoxia induction. Then, we evaluated the impact of vitamin C treatment on the gene expression level of four stemness-related genes in hypoxic MCF7 cells.

**Results:**

Our results indicate that vitamin C could reduce proliferation and stemness states in CSCs possibly by induction of apoptotic markers such as *BAX*, along with attenuating stemness markers, including *NF-κB1*, and *DNMT1* gene expressions.

**Conclusion:**

According to our findings, vitamin C administration would become a new approach to avoiding the stimulation of CSCs during cancer therapies.

## Introduction

 Cancer stem cells (CSCs) are a smaller group of tumor cells that are responsible for cancer development, progress, recurrence, and metastasis. This can be attributed to several features, including self-renewal, unlimited proliferation potency, and invasion and migration potency.^1,2^ CSCs are relatively resistant to conventional chemotherapy, thereby there is a need to develop novel approaches to eradicate CSCs from tumor niche.^3^ It is possible for cancer to relapse if CSCs are not cleared. Therefore, targeting the CSCs is in demand for developing a successful cancer therapeutic regimen.

 Based on clinical findings, one of the indications related to poor prognosis and metastasis is tumor hypoxia,^4^ which can promote resistance to chemo and radiotherapeutic agents.^5,6^ Moreover, recent researches indicate that in many human cancers, hypoxic niche displays a crucial impact in the evolution of CSCs, becoming an imperative focus for studying tumor malignancy.^7^ In this regard, previous studies provided evidence that hypoxia increases breast cancer stem cells (bCSCs) population in a hypoxia-inducible factor-1 (*HIF-1*) dependent manner.^8-10^ In other similar studies, it is proposed that hypoxia provokes bCSCs enrichment by m6A-demethylation of *NANOG* mRNA via RNA demethylase *ALKBH5*^11^ or adenosine receptor 2B expression (*A2BR*) following *HIF-1* induction.^12^

 A number of evidence declare the beneficial effect of antioxidants in cancer therapy. Vitamin C is an antioxidant that prevents oxidative damage to cells by inhibiting free radicals production. Consistent with this notion, a high dose of vitamin C has been considered as a therapeutic potential in malignancies.^13,14^ The anti-cancer activity of vitamin C is possibly mediated by redox mechanism, co-factor activity,^15^ and apoptosis induction.^16^ Furthermore, it has been revealed that a high dose of vitamin C triggers DNA damage in CSCs and upregulates epigenetic demethylases that eventually reverse CSCs phenotypes.^13,15,17^

 This research is thus compiled to analyze the impact of the higher doses of vitamin C on the expression level of *HIF-1α*, *NF-κB1*, *BAX,* and *DNMT1* genes in MCF7 cells undergoing hypoxia, as an inducer of CSCs characteristics; investigating the probability that high dosages of vitamin C can inhibit tumor recurrence by eradicating CSCs.

## Materials and Methods

###  Cell culture

 MCF7 cell line (purchased from Pasteur Institute; NCBI code: C135) was cultured in RPMI1640 medium (Sigma), supplemented with 10% Fetal Bovine Serum (Gibco), and 1% Pen-Strep solution. Cultured cells were kept in a humidified incubator that provided 95% O2 and 5% CO2 at 37 °C. Cells were regularly passaged every three days and all assays were carried out when cells were sub-confluent. To perform hypoxic exposure, hypoxia was imitated using a humidified gas mixture of 94% N2, 5% CO2, and 1% O2 at 37 °C.

###  Vitamin C preparation

 A stock solution containing 0.2 M (0.035 g/mL) of vitamin C (Sigma) was prepared by dissolving it in dimethyl sulfoxide (DMSO, Merck). Then a different concentration of the working solution was prepared by dilution of the stock solution with RPMI-1640 immediately before use.

###  Cell viability assay

 To study how vitamin C affects cell viability, an MTT assay was conducted.^18^ In brief, 1 × 10^4^ cells/well were seeded in a 96-well plate and cultured for 24 hours in 10% FBS RPMI1640 medium. After 24 hours, variable concentrations of vitamin C (2.5-50 mM) were added to each well and cell plates were incubated in the same medium containing 2% FBS for 24 hours, 48 hours, and 72 hours. Following this, 20 mL of MTT solution (5 mg/mL) was loaded into each well for 4 hours (Sigma). After removing the media containing MTT, 200 μL of DMSO solution was added to solubilize the formazan crystals. The absorbance rate was then determined at 570 nm with a microplate reader (BioTek). The experiments were done in triplicate and the relative viability was calculated relative to the control cells (percentage of control).

###  Magnetic-activated cell sorting assay

 To verify whether hypoxic conditions increase the number of CSCs, we isolated and enriched CD44^+^ and CD24^-^ cells from hypoxic MCF7 cells using magnetic-activated cell sorting (MACS). After dissociating adherent cells with 0.25% trypsin-EDTA (Gibco), the cells were washed and resuspended in PBS. Next, 1 × 10^7^ cells were incubated with 20 μL anti-CD44 MACS microbeads at 4 °C for 15 minutes. After resuspending the cells in PBS and Miltenyi buffer (500 μL), the cells passed through LS positive selection column in the presence of a magnetic field, so that the CD44^+^ cells remained in the column. Then CD44^+^ cells were obtained after the magnetic separator was removed from the column and the cell culture was washed with Miltenyi buffer. A subsequent experiment involved resuspending 1 × 10^7^ CD44^+^ cells in 40 μL of buffer and adding 10 μL of monoclonal CD24 antibody conjugated to biotin and incubating at 4 °C for 15 minutes. Following washing and centrifugation, the cells were resuspended in Miltenyi buffer and 20 μL Anti biotin-CD24 microbeads for 15 minutes at 4 °C. Following the washing of cells in PBS and resuspending in Miltenyi buffer, CD24^-^ cells were passed through the column and collected. Obtained cells were CD44^+^/CD24^-^ cells.

###  Flow cytometric analysis

 Briefly, three cultured groups of MCF7 cells (normoxic, hypoxic, and vitamin C-treated hypoxic cells) were detached and washed two times with PBS. The cells (10^6^ in 1% bovine serum albumin in PBS) were incubated with 10 μL of FITC-CD24 and PE-CD44 antibodies (Miltenyi Biotec) at 1/100 dilution at 4 °C for 30 minutes in the dark. The CSCs populations were then isolated based on the CD44^+^/CD24^-^ markers, applying FACSCalibur flow cytometer (BD Bioscience). The results were evaluated using FlowJo Software. Nonspecific results were discovered by proper isotype-matched antibodies.^19,20^

###  RNA isolation, reverse–transcription, and real-time PCR 

 In brief, 2 × 10^6^ cells were treated and collected from each group to test the impacts of vitamin C administration on the expression of cancer stemness-associated genes. Using an RNA extraction kit (Yekta Tajhiz Azma, Iran), RNA was extracted and reverse transcripted (0.5 μg RNA) to cDNA. The real-time PCR was conducted by applying QuantiTect SYRB Green dye (TakaRa) and a Corbett Rotor-Gene^TM^ 6000 HRM system. The target genes were normalized to the reference gene *GAPDH*, and the data were presented as the relative fold difference between the cDNA of the study and the calibrator samples using the ΔΔCT method. All experiments were carried out in triplicate. Real-time PCR primers were specifically designed to span an exon-exon junction or be separated by at least one intron on the corresponding genomic DNA to only amplify the mRNA sequences (Table 1). In addition, to verify primer amplification, PCR products were visualized on 2% agarose gel (Cinnagen).^21^

**Table 1:** Sequences of primers used for real-time PCR analysis

**Genes**	**Primer sequences**	**Product size (bp)**
*GAPDH*	F: 5'-TTGACCTCAACTACATGGTTTACA-3' R: 5'-GCTCCTGGAAGATGGTGATG-3'	126
*HIF-1 α*	F: 5'-TAGCCGAGGAAGAACTATGAAC-3' R: 5'-ACTGAGGTTGGTTACTGTTGG-3'	101
*NF- κ B1*	F: 5'-CAATCATCCACCTTCATTCTCAAC-3' R: 5'-CCACCACATCTTCCTGCTTAG-3'	147
*BAX*	F: 5'-TCAGGATGCGTCCACCAAGAAG-3' R: 5'-TGTGTCCACGGCGGCAATCATC-3'	103
*DNMT1*	F: 5'-GCGGCTCAAAGATTTGGAAAGA-3' R: 5'-CAGGTAGCCCTCCTCGGAT-3'	160

###  Statistical analyses

 The data of independent tests were shown as mean ± standard deviation (SD). To find out whether the results are significant, data analysis was performed by ANOVA and Tukey’s post hoc test in GraphPad Prism version 7.0 (GraphPad Software Inc.). *P* value < 0.05 was considered statistically significant.

## Results and Discussion

###  Effect of vitamin C on the viability of MCF7s

**Figure 1:**
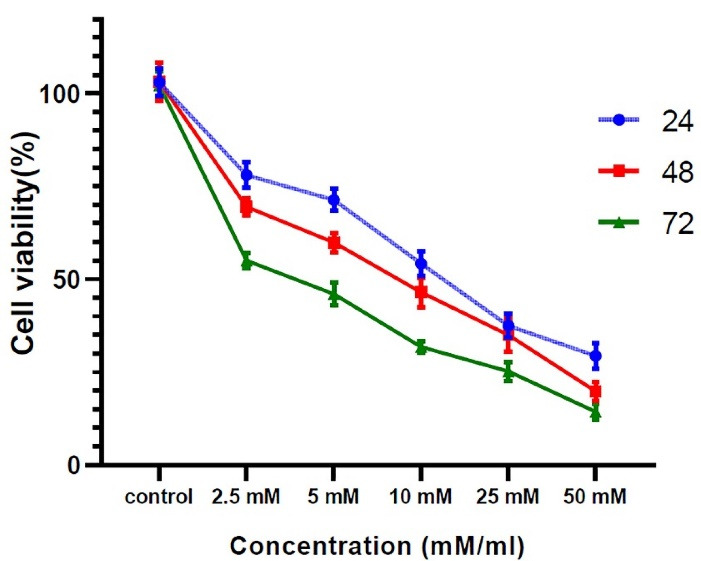
The effect of vitamin C on MCF7s survival rate. MCF7 cells showed the highest survival rate after being exposed to 2.5 mM vitamin C for 24 hours. Contrary, treatment of cells with the dose of 25 and 50 mM leads to cell viability below 50 percent. So, three concentrations of 2.5, 5, and 10 mM were used for the following processes. All experiments were performed in triplicate and data were expressed as mean ± SD

 MCF7 cells were incubated with different concentrations of vitamin C ranging from 2.5 to 50 mM. The cells exhibited different growth rates in various concentrations of vitamin C (Figure 1). As shown in Figure 1, as the concentration of vitamin C increased, the viability of MCF7s decreased. In addition, these results indicated that incubation of MCF7s with concentrations of 25 and 50 mM of vitamin C displayed a promising cytotoxic effect. Accordingly, vitamin C at a concentration of 2.5, 5, and 10 mM was selected for subsequent analyses, for 24 hours.

###  Effect of hypoxia on breast cancer stem cell population in MCF7 cells

 To induce CSCs enrichment in MCF7 cell culture, the cells were kept in a hypoxic condition. Characterization of CSCs in MCF7 cells was verified according to the expression of both CD24 and CD44 markers. Immunofluorescence staining on MACS-isolated cells from normoxic and hypoxic MCF7 cells revealed that the percentage of the positive cells for the CD44 marker and negative cells for the CD24 marker was increased in cells cultured under the hypoxic condition when compared to MCF7 cells without hypoxia pre-treatment (32.9% versus 18.6%) (data are not shown).

###  Effect of vitamin C on breast cancer stem cells during hypoxia induction

**Figure 2:**
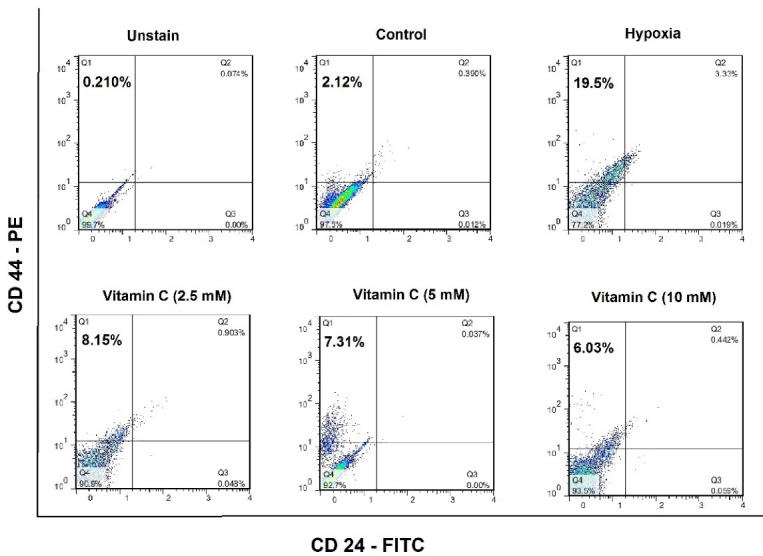
Cancer stem cells (CSCs) population in the normoxic, hypoxic, and vitamin C-treated MCF7 cell line. MCF7 cells were labeled with FITC-conjugated anti-CD24 and PE-conjugated anti-CD44 antibodies, and then the cell populations for each group of control, hypoxia and Vitamin C-treated ones, were characterized according to the surface markers using a FACSCalibur flow cytometer (BD Bioscience, USA). The data were collected and analyzed using FlowJo Software.

**Figure 3:**
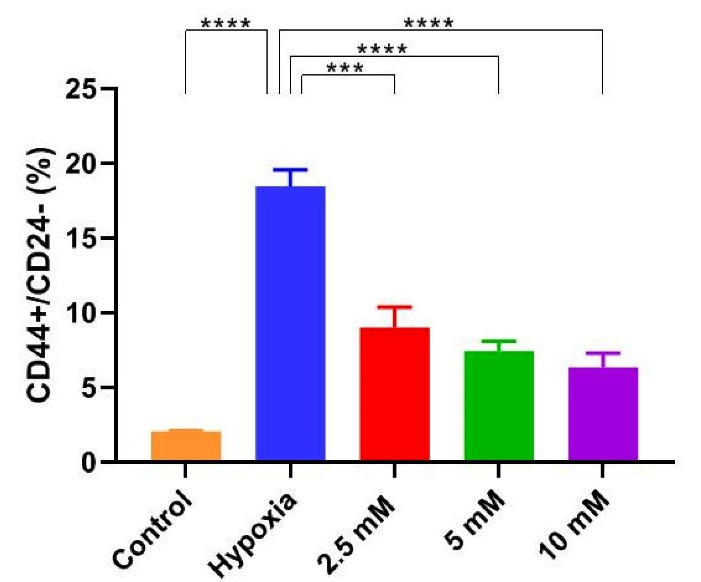
Vitamin C reduced the breast Cancer stem cells (bCSCs) population during hypoxia induction. CD24^-^/CD44^+^ cells as an indicator of bCSCs were stained using FITC-conjugated anti-CD24 and PE-conjugated anti-CD44 antibodies, and analyzed by Flow cytometry in hypoxic MCF7 cells, that were incubated for 24 hours in medium containing three concentrations of 2.5, 5 and 10 mM vitamin C. Data are expressed as mean ± SD of three independent experiments. Statistically significant differences are indicated as *** P < 0.0002 and ****P < 0.0001.

 Flow cytometric data revealed that vitamin C could reduce the number of bCSCs (Figure 2). Compared with untreated hypoxic cells, a significant reduction was found in the level of CD24^-^/CD44^+^ cells in the group receiving vitamin C (Figure 3). These results imply that vitamin C can potentially reduce the rate of bCSCs in human MCF7s during hypoxia induction.

###  Effect of vitamin C on the gene expression level of NF-κB1, BAX, HIF-1α, and DNMT1 genes

**Figure 4:**
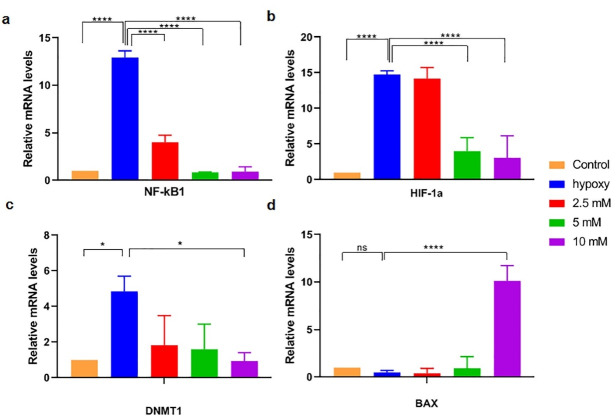
mRNA expression of NF-κB1 (a), HIF-1α (b), DNMT1 (c), and BAX (d), in hypoxic MCF7 cells was carried out by real-time RT-PCR and compared with that expression in normoxic MCF7cells after incubation of the cells with vitamin C (2.5, 5, and 10 mM) for 24 hours. Results are presented as mean ± SD of three independent experiments. Statistically significant differences are indicated as * P < 0.05 and **** *P* < 0.00001.

 In order to assess the impact of vitamin C on gene expression of *NF-κB1*, *BAX*, *HIF-1α,* and *DNMT1*, real-time RT-PCR was carried out. *NF-κB1* mRNA showed an almost 12-fold higher expression level in hypoxia-induced bCSC cells as compared to normoxic MCF7 cells, reaching the lowest level at 24 hours incubation with vitamin C (10 mM) (Figure 4a).

*HIF-1α* and *DNMT1* mRNA expression levels in hypoxic bCSC were also significantly increased in CSCs than that level in normoxic MCF7 cells (respectively 4.8 and 14.7 fold changes; *P* < 0.05) and decreased the following incubation with vitamin C (respectively reached to 0.95 and 3 fold changes; *P* < 0.05) (Figure 4b, c).

 Regarding mRNA expression of *BAX*, vitamin C-treated cells with a dose of 10 mM could significantly increase the gene expression level up to 10-fold, as compared to the control and hypoxic MCF7 cells (Figure 4d).

 As evidenced, CSCs have the intrinsic capacity for self-renewal and differentiation. These cells are known to be the origin of most cancer cells and are responsible for tumor resistance and relapse. It has been reported that CSCs possess elevated protection levels against oxidative stress which was induced by reactive oxygen species compared with non-stem-like cancer cells.^22,23^

 Evidence indicates that hypoxia, the major feature of solid tumors, enhances the proportion of CSCs in a HIF-1 dependent way.^9,12,24^

 Vitamin C, a prominent antioxidant, has a binary function in CSCs dynamics by scavenging free radicals and protecting cells from oxidative damage.^25,26^ Beyond the protective effect, vitamin C has also cytotoxic effects on cancer cells that are mediated by increasing the ROS levels and impeding the homeostasis of energy at high concentrations.^27^ Contrary to low vitamin C concentrations (5–25 μM), which leads to CSCs proliferation,^27^ higher concentrations (10 g/d) are conceivably toxic to cancerous cells compared to healthy cells.^15,28^

 In this study, we considered the impact s of vitamins C at three concentrations of 2.5, 5, and 10 mM on the genes expression level of *NF-κB1*, *HIF-1α*, *BAX,* and *DNMT1* in the MCF7 cells undergoing hypoxia, as an inducer of CSCs characteristics.

 The molecular pathways needed for CSCs preservation are explained in several studies. Among them, *NF- κB* is the known transcription factor with pivotal effects on cell survival, immunity, and inflammation. New findings show that mammalian *NF- κB* controls the self-renewal of bCSCs in a Her2-dependent manner.^29^ Deregulation of *NF- κB* activity leads to the constant nuclear localization of p65, p52, p50, RelB, and cRel, resulting in the up-regulation of anti-apoptotic factors and the interference with cell proliferation and death balance in the following.^30,31^ It is also revealed that activation of *NF- κB* by inflammatory cytokines or epigenetic dysregulation could stimulate NOTCH signaling pathway in CSCs, increasing CSC populations. Therefore, *NF- κB* activation plays a critical role in regulating of CSCs populations.^32^ In this way, it is reported that small molecules like parthenolide, pyrrolidine dithiocarbamate, and its analog diethyldithiocarbamate could target bCSCs.^29^ It is also evident from in vitro studies that curcumin and epigallocatechin gallate diminished the stemness characteristics of the breast cancer cells by adjusting *STAT3–NF- κB* signaling pathways.^33^

 In our study, the *NF-κB1* mRNA content in hypoxic MCF7 was significantly higher than that level in normoxic MCF7 cells which was significantly decreased following incubation with vitamin C for 24 hours. Our finding is in line with the prior research indicating that vitamin C can inhibit the *NF- κB* activation in a dose-dependent way in addition to inhibited TNFα-induced degradation of IkBcα.^34^

 In some cancers, growing data indicates that hypoxia plays an important function in CSCs expansion,^23^ and oxygen-dependent transcription activators such as hypoxia-inducible factors (HIFs), are the major mediators of cell response to low oxygen status.^35,36^ These factors mediate tumor adaption with stressful situations, leading to various gene transcriptions which take part in glycolysis, angiogenesis, metastasis and resistance to radio and chemotherapy.^37,38^ Enhanced activity of HIF in hypoxic situations is correlated with an enhanced level of antioxidant production which is necessary for maintaining redox homeostasis and promoting the appearance of stem cell properties in breast cancer.^37,39^ These events lead to poor outcomes in a range of cancers. As a result, now HIFs are thought to be a key goal for cancer treatment. It is worth noting that vitamin C, as a cofactor is required for hydroxylation reactions which can control the activity and stability of HIFs α subunits.^40^ Accordingly, supplementation of cancer cells with increased vitamin C concentration could promote the hydroxylation and then reduce the activity of the HIFs, thus attenuating tumorigenesis.^41,42^ In the present research in order to evaluate the effect of vitamin C in the process of the bCSC-like cells, the relative mRNA expression of *HIF-1α* was analyzed. The finding showed that the expression level of the *HIF-1α* gene was decreased significantly in hypoxic-treated cells in relation to non-treated hypoxic cells. This finding is inconsistent with earlier studies showing that vitamin C decreased *HIF-1α* levels in a dose-dependent pattern.^40,43^

 To clarify the impact of high-dose vitamin C in apoptotic events, we evaluated *BAX* pro-apoptotic gene expression. Our data showed that vitamin C at 10 mM concentration could significantly enhance the expression level of *BAX*. In this context, it is noteworthy to highlight that Bax activity is counteracted by Bcl-2, an apoptosis-promoting protein. It was demonstrated that produced ROS by a high dose of vitamin C induces programmed cell death in bCSCs. These results imply that a higher concentration of vitamin C, 10 and 20 mM, results in several events in bCSCs as follows: cell damage by enhancing the ROS level, mitochondrial damage by induction of oxidative stress, and intrinsic apoptosis pathway.^28^

 In addition, the role of vitamin C in the epigenetic control of gene expression has received growing notice in many backgrounds, from the normal performance of cells to cancer therapy.^26,44,45^ It was determined that vitamin C acts as a cofactor for methylcytosine dioxygenases which acts as DNA demethylase and some JmjC domain-containing histone demethylases.^46^ Here, we examined the effect of vitamin C on the gene expression level of *DNMT1*. Previous data support a crucial role for* DNMT1 *in the tumorigenic phenotype of CSCs and show that suppression of DNMT activity reverses the atypical self-renewal characteristics of CSCs.^47^ Based on our results vitamin C treatment with the dose of 10 mM significantly reduces the *DNMT1* gene expression level compared to non-treated hypoxic MCF7 cells.

 Overall, our results indicate that vitamin C can reduce proliferation and stemness states in CSCs possibly through the induction of apoptotic markers such as *BAX*, along with attenuating stemness markers, including *NF-κB1* and *DNMT1* gene expressions.

## Conclusion

 To conclude, a high dose of vitamin C could potentially inhibit CSCs, an aspect of the new medical trend of targeting CSCs. Despite the limitation of the study, our findings in concordance with the recent studies^28,48^ revealed that high doses of vitamin C drive cytotoxicity and gene expression modifications related to the genes that are involved in cancer stemness phenotypes. Therefore, the pharmacological dose of vitamin C (~ > 10 mM) could be possibly applied as a hopeful future therapeutic adjuvant, particularly in higher stages of breast cancer, and needs confirmation pre-clinically.

## Acknowledgments

 The authors greatly appreciate the Drug Applied Research Center of the Tabriz University of Medical Science for supporting this project. (Grant number: 5/104/672).

## Competing Interests

 The authors declared no conflicts of interest.

## Ethical Approval

 Not applicable.
